# A Case-Control Study on the Risk Factors for Meningococcal Disease among Children in Greece

**DOI:** 10.1371/journal.pone.0158524

**Published:** 2016-06-28

**Authors:** Christos Hadjichristodoulou, George Mpalaouras, Vasiliki Vasilopoulou, Antonios Katsioulis, George Rachiotis, Kalliopi Theodoridou, Georgia Tzanakaki, Vassiliki Syriopoulou, Maria Theodoridou

**Affiliations:** 1 Department of Hygiene and Epidemiology, Faculty of Medicine, University of Thessaly, Larissa, Greece; 2 Aghia Sophia Children’s Hospital, National and Kapodistrian University of Athens School of Medicine, Athens, Greece; 3 National Reference Centre for Meningitis, National School of Public Health, Athens, Greece; Universidad Nacional de la Plata, ARGENTINA

## Abstract

**Purpose:**

The aim of this study was to identify environmental or genetic risk factors that are associated with invasive meningococcal disease (IMD) in children in Greece.

**Methods:**

A case-control study was performed in 133 children (44 cases and 89 controls) aged between 0–14 years, who were hospitalized in a children's hospital in Athens. Demographics and possible risk factors were collected by the use of a structured questionnaire. To investigate the association of mannose binding lectin (MBL) with IMD, a frequency analysis of the haplotypes of the *MBL2* gene and quantitative measurement of MBL serum protein levels were performed using Nanogen NanoChipR 400 technology and immuno-enzyme techniques, respectively.

**Results:**

The multivariate analysis revealed that changes in a child's life setting (relocation or vacation, OR = 7.16), paternal smoking (OR = 4.51), upper respiratory tract infection within the previous month (OR = 3.04) and the density of people in the house/100m^2^ (OR = 3.16), were independent risk factors associated with IMD. Overall 18.8% of patients had a *MBL2* genotype with low functionality compared to 10.1% of healthy controls, but this was not statistically significant (p = 0.189).

**Conclusion:**

Prevention strategies aimed at reducing parental smoking and other risk factors identified in this study could decrease the risk of IMD among children in Greece.

## Introduction

Invasive meningococcal disease (IMD) is a contagious bacterial disease caused by a meningococcus (*Neisseria meningitidis*), a Gram-negative bacterium that is classified into 13 capsular groups according to its capsular polysaccharides. Six of these (A, B, C, Y, X and W), are of clinical significance as they cause invasive infections. In Europe, groups B and C are mainly responsible for IMD [1]. In the USA, groups B, C and Y cause a high proportion of IMD [2], while in Africa group A is predominant and groups W, X and C are also endemic [3]. Meningitis and septicemia are the two main clinical forms of IMD, and sometimes both clinical forms are found in the same patient. Meningococcal meningitis is a serious infection of the meninges that can cause severe brain damage and other sequelae. In meningococcal septicaemia, the onset of the symptoms is sudden and death can follow within hours. IMD has a high fatality rate and many survivors develop permanent sequelae [4–5].

Meningococcal infections are transmitted between people through respiratory droplets or secretions. *N*. *meningitidis* inhabits the mucosal membrane of the nose and throat, where it usually causes no harm [6–7]. There is substantial evidence that approximately 10% of the general population are asymptomatic carriers, although this rate varies with age, and is associated with a peak in early adulthood [8–10]. Several polysaccharide and conjugate vaccines are available for the protection of humans from the most common capsular groups of IMD. Polysaccharide vaccines are available in bivalent (A, C), trivalent (A, C, W), and quadrivalent (A, C, W, Y) forms. Conjugate vaccines, which are more immunogenic and can provide herd immunity, are available in monovalent (A or C), quadrivalent (A, C, W, Y), or combinatorial (group C and *Haemophilus influenzae* type b) forms [10]. Recently, a new vaccine against group B has been developed based on reverse vaccinology [11]. In Greece, the vaccine against group C has been included in the National Vaccination Programme (NVP) since 2006; recently, a vaccine against group B was made available in Greece but is not yet included in the NVP.

The risk of meningococcal infection in an individual is dependent on the balance of the virulence of the strain and the host’s immune response. Moreover, several environmental risk factors have been associated with the disease in several countries [12–13]. Both active and passive smoking in particular have been found to increase the risk of IMD in pediatric populations [14–16]. Other risk factors include crowded living conditions, close contact with an infected person, a history of recent upper respiratory tract infections and low socio-economic status [17–20]. Finally, individual risk factors such as an underlying disease (e.g., malignancies) or asplenia are also associated with a higher risk of developing IMD [21–22].

Genetic mutations of MBL(an acute protein phase that contributes to the elimination of bacteria by activating the complement system) have been identified as possible risk factors associated with IMD in several studies [23–25]. As contradictory results exist regarding the role of MBL in IMD, and there is no available evidence from Greece, we decided to investigate the role of MBL further as a predisposing factor for IMD. The aim of the current study was therefore to identify possible environmental or genetic factors that increase the predisposition of children in Greece to developing IMD, including an analysis of MBL serum protein levels and haplotype analysis of *MBL2* gene.

## Materials and Methods

### Ethics statement

Approval of the study protocol was received by the Ethics Committee of Aghia Sofia Children’s Hospital, which waived the need for written consent. Parents or guardians were informed about the aim of the study, and they provided written consent for their child’s participation in the study.

### Study design

A case-control study was performed using 133 children (44 cases and 89 controls). All participants were children aged between 0–14 years, who had been hospitalized in two children's hospitals (Aghia Sophia and P & A Kyriakou) in Athens, Greece, within a 2-year period from January 2011 to December 2012.

Cases were had been hospitalized with a diagnosis of IMD (meningococcal meningitis and/or sepsis). In all cases, *N*. *meningitidis* was identified in samples of biological material (blood or CSF) in the laboratory and isolated using bacterial cultures or molecular techniques, such as polymerase chain reaction (PCR). In addition, to increase the reliability and the power of our genetic research, the frequency analysis of the gene polymorphisms of *MBL2*, included 45 extra blood samples from the National Reference Centre for Meningitis (NRCM). These samples were collected from cases of IMD in children aged 0–14 years, who had become ill during the same period (2011–2012) and had been admitted to various hospitals across Greece. Our study was designed to encompass two spring seasons in order to capture any respective seasonal increase in IMD.

Controls were children hospitalized in the surgical wards of the two hospitals with a diagnosis that was unrelated to IMD or other infections. All controls were matched to cases using the sex and age (year of birth) of each child, and the week of admission. When it was possible, at least two controls were randomly selected for every case.

### Data collection

In both cases and controls, a whole blood sample (3–4 mL) was collected on the day the child was admitted to the hospital, in order to study the *MBL2* haplotypes and the serum levels of MBL protein. For each blood sample, 0.5 mL was stored immediately at -80°C, in order to be used in the frequency analysis of *MBL2* polymorphisms. After centrifugation of the remaining blood sample, plasma was collected and kept readily cryopreserved at -80°C for the quantitative measurement of MBL.

### Questionnaire

A questionnaire was distributed to the parents of both cases and controls, in order to obtain information on the following: their child’s demographic details (sex, age, race, height, weight, mother’s/father’s educational status, social security status, use of paediatrician or general practitioner services, family income, specific population group); family history (number of family members, number of children, birth order, family medical history, family medical history of meningitis); housing environment (size of the house in square meters, number of household members, heating system, type of house [single family house or block of flats], exposure to passive smoking at home). Moreover, the questionnaire included questions regarding perinatal history and breastfeeding; history of hospitalization; vaccination history; medications/ social history during the last month: attendance at nursery school, elementary/high school/college attendance; participation in sporting events; attendance at parties and playgrounds; church attendance; visits to restaurants or coffee bars; kissing other people; use of public transport; and change of life setting (relocation or vacation). If the answer to the question of change in life setting was positive the parents were asked to specify. Parents were also asked to report if their children had signs and symptoms compatible with upper respiratory infection (fever and/or cough and/or sore throat and/or rhinitis) during the last month. It should be noted that some questions in the questionnaire (e.g., kissing other people and coffee bars attendance) were relevant to older children (≥12 years old), while other questions (e.g., nursery and play areas attendance) were relevant to children aged ≤10 years.

### Genetic analysis of MBL

*MBL2* includes three polymorphic sites in exon 1 (cd52, cd54, cd57) forming the haplotypes AO, OO, and AA, three polymorphisms in the area of the promoter (-550, -221, +4) forming the haplotypes HY, LY and LX and four polymorphic sites located in exon 4 (carbohydrate recognition domain-CRD). The frequency analysis of *MBL2* polymorphisms was performed by the Choremeio Research Laboratory of Medical Genetics at the University of Athens. Of the 44 cases of IMD, only 36 agreed to participate in the genetic analysis while 69 out of the 89 of the controls agreed to participate (response rates of 81.8% and 77.5%, respectively). To increase the power of the study, 45 cases of IMD from NRCM were included in the genetic analysis to achieve a total number of 81 cases of IMD analyzed.

Genomic DNA was isolated from 350 μl of peripheral blood, using the BioRobotR M48 System (Qiagen, Hilden, Germany) and the MagAttractR DNA Blood Midi M48 Kit (Qiagen, Hilden, Germany). For the characterization of the six SNPs in *MBL2*, we developed an advanced high throughout methodology using the Nanogen NanoChipR 400 system (NC400, Nanogen Inc). Three separate regions of the *MBL2* gene containing SNPs were designed for PCR amplification using the PrimerQuestSM tool provided by IDTR (Integrated DNA Technologies). The set-up of all PCR reactions was performed automatically using the Biorobot 3000 platform (Qiagen, Hilden, Germany) and carried out in a Techne TC-412 thermal cycler using the amplicon-down format of the Nanogen protocol. Results were estimated by the instrument's software.

The quantitative measurement of the MBL levels of serum was performed in 43 of 44 (97.7%) patients, and in 73 of 89 (82.0%) controls at the Department of Hygiene and Epidemiology at the University of Thessaly, using the MBL Oligomer ELISA Kit (BioPorto Diagnostics Co.).

### Statistical analysis

All data collected from the study participants (questionnaire, clinical and laboratory results) were entered into an electronic database using Epi-info software (version 3.5.3, CDC, Atlanta). Statistical analysis was performed using IBM SPSS Statistics software (v.22.0. Armonk, NY: IBM Corp.).

Quantitative variables were presented either as mean values with standard deviation or as a median value with the interquartile range (IQR). Qualitative variables were presented as absolute and relative frequencies with the corresponding 95% confidence intervals (95% CI). The receiver operating characteristic (ROC) curve analysis was conducted to determine the optimal cutoff values of the quantitative variable density (number of persons per 100 m^2^), which was used to distinguish between IMD cases and controls.

In the univariate analysis, the Chi-square test or the Fisher's exact test was used to investigate the associations between the qualitative variables. The Chi-square test for trend was used to assess any dose response relationship between the ordinal factors (e.g., number of cigarettes per day) and IMD. The Mann-Whitney test was used to explore differences between IMD cases and controls with regards to quantitative non-normally distributed variables.

In the multivariate analysis, multiple logistic regression analysis was performed, using the backward stepwise conditional method with a removal criterion of p-value equal to or greater than 0.10, inorder to identify the independent risk factors for the onset of IMD by calculating the odds ratios (ORs) and the corresponding 95% CI. The dependent variable was IMD and as independent variables were used all the statistically significant risk factors found in the univariate analysis together with age and gender were used as independent variables. For the genetic analysis of MBL, the necessary sample size was calculated to be 200 children (100 per group) assuming a study power of 0.80, alpha 0.05, and considering 10.0% of low MBL in controls to identify a significant OR 3.00 (cases vs. controls). A result with a p-value <0.05 was considered to be statistically significant.

## Results

### Participant characteristics

During the initial study period, 50 cases of IMD were hospitalized in both hospitals. In two cases, the diagnosis was not confirmed and the guardians of four patients (all females) refused to participate in the study. Overall, 44 cases of IMD participated in the study (response rate: 91.1%), of whom 27 (61.3%) were males and 17 (38.7%) females. The median age was 3 years (IQR: 1–4 years). Regarding race, 93.2% of them were white, 2.3% black, and 4.5% were from an other racial background. Also, 18 IMD cases (~41%) were second-generation immigrants, of whom 13 (~72%) were born in Greece while their parents were from Albania. Seven patients presented with septicemia, nine had meningitis and 28 patients had both. Sixteen patients were diagnosed using PCR and 28 through culture tests. The majority of cases were caused by group B meningococcus (36/44), one patient had group A and one had group W, while meningococcus was nongroupable in six patients. Furthermore, 23 out of 40 (57.5%) cases of IMD had been vaccinated with meningococcal meningitis C vaccine.

Regarding the controls, 99 patients were initially recorded but only 89 controls participated in the study (response rate: 89.9%). Out of these 89 controls, 65 (73%) were males and 24 (27%) were females, while 98.8%were white, 0% were black and 1.2% had another racial background. The median age of controls was 3 years (IQR: 1–5 years). Approximately 24% of the controls were second-generation immigrants.

### Univariate analysis

All statistically significant risk factors, according to the univariate analysis, are presented in Table 1. None of the other parameters included in the questionnaire, including gender, age, race, pets, normal birth, breastfeeding insurance status, annual family income, parental education level, maternal smoking, maternal cigarettes consumption per day, fever, cough, headache, vaccination status for *N*. *meningitidis* type C, or parental occupation, were found to have any statistically significant association with the occurrence of IMD. Moreover, factors such as the use of antibiotics, participation in sports activities, participation in parties, or kissing other people during the previous month were not statistically significant. Finally, no statistically significant difference was found in the serum protein levels of MBL between cases and controls.

**Table 1:** Statistically significant risk factors for invasive meningococcal disease (univariate analysis).

Variables	Cases (%)	Controls (%)	p-value*
Immigrant	18/44 (40.9%)	20/84 (23.8%)	0.044
Monitoring child’s health by the same pediatrician	29/42 (69%)	72/84 (85.7%)	0.027
Father smoker	30/42 (71.4%)	36/82 (43.9%)	0.004
Number of cigarrets per day (fathers)			
(≥20)	26/42 (61.9%)	30/82 (36.6%)	0.004**
(1–19)	4/42 (9.5%)	6/82 (7.3%)	
Density: number of people/ house dimensions (100 m^2^) median value (IQR)	5.3 (4.0–6.7)	4.2 (3.2–5.6)	0.019***
Density: ≥ 4.4 (number of people per 100 m^2^ house)	25/37 (67.6%)	33/73 (45.2%)	0.026
Contact with cases of IMD	4/41(9.8%)	0/84 (0%)	0.010
History of viral respiratory infection	22/43 (51.2%)	27/84 (32.1%)	0.037
Sore throat	8/43 (18.6%)	4/84 (4.8%)	0.021
Church attendance	12/43 (27.9%)	40/82 (48.8%)	0.024
Relocation or vacation during the previous month	10/43 (23.3%)	6/83 (7.2%)	0.010

*Chi-square test or Fisher’s exact test

** Chi-square test for trend

***Mann-Whitney test.

As shown in Table 1, a change in life setting in the previous month (vacation or relocation) was identified as a risk factor (p = 0.010). The most frequent answer within the positive responses in the relocation/vacation question among cases, was vacation to another town within Greece (60%) or abroad (20%) for ≥5days. Moreover, 20% reported a relocation without specifying whether it was in the same town or in another town. Finally, it should be noted that most of the vacations were to celebrate Easter, or to visit relatives and friends in the migrants’ country of origin (mainly Albania).

Parental smoking (p = 0.030, OR = 2.48) and paternal smoking (p = 0.004, OR = 3.19) were identified to be associated with IMD, although the difference for maternal smoking was not statistically significant (p = 0.150, OR = 1.74). Moreover, for fathers a dose-response was revealed between number of cigarettes per day and therisk of IDM. The OR for smoking ≥20 cigarettes per day was 3.32 (95% C.I.: 1.46–7.58), compared to 2.56 (95% C.I.: 0.62–10.53) for smoking 1–19 cigarettes per day. The mean number of cigarettes per day smoked by the fathers was double of that of the mothers (20 vs. 10).

### Multivariate analysis

As shown in Table 2, the multivariate analysis revealed the following independent risk factors: relocation or vacation during the last month (OR = 7.16; 95% CI: 1.80–28.50), paternal smoking (OR = 4.51; 95% CI: 1.60–12.67), recent history (past 30 days) of viral respiratory infection (OR = 3.04; 95% CI: 1.17–7.91) and crowd density at home (OR = 3.16; 95% CI: 1.16–8.60).

**Table 2:** Multivariate analysis (logistic regression) of the risk factors for invasive meningococcal disease.

Risk factors*	OR	95% C.I.	p-value
Father smoker	4.51	1.60–12.67	0.004
Recent symptoms of viral respiratory infection	3.04	1.17–7.91	0.023
Relocation or vacation during the previous month	7.16	1.80–28.50	0.005
Density: ≥ 4.4 (number of people per 100 m^2^ house)	3.16	1.16–8.60	0.024

*Adjusted for age and gender.

### *MBL2* haplotype analysis

According to the analysis of the MBL polymorphisms in exon 1 and in the promoter region, the patients were classified into three groups of high (HYA / HYA, HYA / LYA, LYA / LYA, and LYA / LXA), moderate (LXA / LXA, HYA / O and LYA / O) and low (LXA / O and O / O) functionality of the *MBL2* gene, except for one sample. The low functionality of *MBL2*, was observed in 18.8% of the patients and in 10.1% of the control group, but this difference was not statistically significant (p = 0.189) (Table 3).

**Table 3:** Cases/Controls classification according to MBL genotype.

MBL2 gene functionality	Cases^+^	Controls	Total
	N (%)	N (%)	N (%)
**High**	44 (55)	43 (62.3)	87 (58.4)
**Moderate**	21 (26.3)	19 (27.5)	40 (26.8)
**Low**	15 (18.8)	7 (10.1)	22 (14.8)
**Total**	80	69	149

^**+**^ One case was not possible to be classified by MBL2 gene functionality

p-value * = 0.189, *Chi-square test for trend.

## Discussion

This study revealed that a recent history of relocation or holiday, paternal smoking, a recent history of viral upper respiratory tract infection and crowded home conditions were independent risk factors for IMD. One interesting and possibly important novel finding of our study was the association between a change in the life setting either by relocation or vacation in the previous month with IMD (OR = 7.16). During these trips, extensive social activities are expected together with changes in pharyngeal flora as indicated by previous studies [26]. The precise mechanism by which travelling for a vacation could be a risk factor is unknown, but a recent case-crossover study reported that travelling abroad was independently associated with meningococcal carriage [27]. Relocation/vacation activities could be linked with changes in the flora of the nasopharynx as a result of environmental changes, including colonization by strains of *N*. *meningitidis*. It is known that the risk of invasive IMD is higher in newly colonized people [28]. This finding could have implications related to the systematic vaccination of unvaccinated young people who will be travelling or relocating. This prevention activity has a number of practical limitations related to the cost of the vaccine, the need for booster doses and the time needed to achieve protective immunity. Moreover, as reported by Tzanakaki et al., the suggested coverage of the 4CmenB vaccine would be 89% in Greece [29].

Our findings are in line with well-known risk factors for IMD, such as a history of viral respiratory tract infections [30], and parental passive smoking. A stronger association between paternal smoking together with a dose–response effect has been identified previously, while in a previous study maternal smoking was identified as more important risk factor [31]. The most plausible explanation for our findings was the fact that the fathers in our study smoked a higher average number (20 per day) of cigarettes compared to mothers (10 per day), while both parents contribute almost equally to child care. A meta-analysis found a significant association, between passive smoking and IMD in children [32]. In addition, a positive, statistically significant association between passive smoking and the carriage of *N*. *meningitidis* has also been documented in previous studies. It has been suggested that this increased risk may be attributed to the increased ability of the bacteria to adhere to mucosa in the presence of smoke [33].

Finally, multiple logistic regression analysis has revealed that the crowd density in the house (number of persons per 100 square meters of the house) was an independent risk factor for IMD. It should be noted that a nationwide population-based case-control study among preschool children reported that the risk of IMD increased with an increasing household density [34]. A number of studies have demonstrated a positive correlation between low socio-economic status and the risk of invasive meningococcal infections [17–20]. For this purpose, increased population density in the household has been considered as an indirect indicator of low socio-economic status.

The present study also attempted to explore the correlation between a particular genotype of the *MBL2* gene and its predisposition to IMD. The patients were classified into three groups of high, moderate and low functionality of the *MBL2* gene, based on the combination of polymorphisms in exon 1 and in the region of the promoter. Patients mostly had the low functionality genotype, compared to healthy controls (Table 3, 18.8% vs. 10.1%, p = 0.189). The difference was not statistically significant, probably because of the relatively small number of cases and controls, which resulted in an underpowered study. Several studies have reported different results regarding the association between the MBL protein and IMD. Summerfield et al. [34] were the first to report the possible link between polymorphisms of the *MBL2* gene and the appearance of unusual and severe infections in adults. Moreover, the correlation between IMD and *MBL2* polymorphisms was found in a case-control study by Hibberd et al. [23]. This study revealed a significantly higher frequency of homozygosity and heterozygosity in patients with IMD compared to healthy controls, which was associated with an increased risk for IMD [23]. The authors estimated that approximately 32% of IMD cases might be attributed to *MBL2* polymorphisms. Other studies supported these findings [24–25]. However, another case-control study including 5500 Europeans (296 case and 5196 controls) questioned the above findings and the authors concluded that there was no correlation between *MBL2* polymorphisms and IMD [35].

Our results are subject to several limitations, with the most significant being the limited number of participants. The study power for the effect of MBL was estimated at 0.31,which was low. Thus, the fact that we did not identify a statistically significant difference does not mean that one does not exist in reality. Another limitation was related to the case-control design. Given the absence of the criterion for a temporal association in the case-control studies, we cannot claim that the associations observed in the present study between several risk factors and the outcome are causative. Moreover, our case-control study design is prone to information bias. In particular, the parents of the cases may report (recall) several exposures more readily than controls. However, on the other hand, it is not likely that parents of cases over-reported their smoking habits, and consequently, the exposure of their children to passive smoking. On the contrary, it would be expected that parents would under-report their smoking activities, which could lead to an underestimation of the impact of parental smoking on the risk associated with IMD. A further limitation of our study was that symptoms that were suggestive of an infection of the upper respiratory tract were based on self-reports, without the implementation of serological tests for the detection of viral antigens. Finally, we matched cases to controls by age and thus we lost the opportunity to verify the age as a risk factor. However, as age is a well-known strong confounder, we preferred to control for age to be able to study the other possible risk factors.

In conclusion, our case-control study indicated that paternal smoking, a recent history of upper respiratory tract viral infection, crowded households and recent relocation/vacation activities were independent risk factors for IMD. Additional studies are needed to explore in detail the role of relocation or holidays as risk factors for IMD and to assess the actual risk posed. Preventive activities aimed at reducing parental smoking and other risk factors could decrease the risk of IMD among children in Greece.
